# Effects of disease on foraging behaviour and success in an individual free-ranging northern elephant seal

**DOI:** 10.1093/conphys/coad034

**Published:** 2023-05-25

**Authors:** Rachel R Holser, Daniel E Crocker, Arina B Favilla, Taiki Adachi, Theresa R Keates, Yasuhiko Naito, Daniel P Costa

**Affiliations:** Institute of Marine Sciences, University of California Santa Cruz, 115 McAllister Way, Santa Cruz, CA, 95060, USA; Department of Biology, Sonoma State University, Rohnert Park, California, 94928, USA; Department of Ecology and Evolutionary Biology, University of California Santa Cruz, Santa Cruz, California, 95064 USA; Department of Ecology and Evolutionary Biology, University of California Santa Cruz, Santa Cruz, California, 95064 USA; National Institute of Polar Research, Tachikawa, Tokyo, Japan; Department of Ocean Sciences, University of California Santa Cruz, Santa Cruz, California, 95064, USA; National Institute of Polar Research, Tachikawa, Tokyo, Japan; Institute of Marine Sciences, University of California Santa Cruz, 115 McAllister Way, Santa Cruz, CA, 95060, USA; Department of Ecology and Evolutionary Biology, University of California Santa Cruz, Santa Cruz, California, 95064 USA

**Keywords:** Pinniped, infection, foraging, energetics, ecophysiology

## Abstract

Evaluating consequences of stressors on vital rates in marine mammals is of considerable interest to scientific and regulatory bodies. Many of these species face numerous anthropogenic and environmental disturbances. Despite its importance as a critical form of mortality, little is known about disease progression in air-breathing marine megafauna at sea. We examined the movement, diving, foraging behaviour and physiological state of an adult female northern elephant seal (*Mirounga angustirostris*) who suffered from an infection while at sea. Comparing her to healthy individuals, we identified abnormal behavioural patterns from high-resolution biologging instruments that are likely indicators of diseased and deteriorating condition. We observed continuous extended (3–30 minutes) surface intervals coinciding with almost no foraging attempts (jaw motion) during 2 weeks of acute illness early in her post-breeding foraging trip. Elephant seals typically spend ~ 2 minutes at the surface. There were less frequent but highly extended (30–200 minutes) surface periods across the remainder of the trip. Dive duration declined throughout the trip rather than increasing. This seal returned in the poorest body condition recorded for an adult female elephant seal (18.3% adipose tissue; post-breeding trip average is 30.4%). She was immunocompromised at the end of her foraging trip and has not been seen since that moulting season. The timing and severity of the illness, which began during the end of the energy-intensive lactation fast, forced this animal over a tipping point from which she could not recover. Additional physiological constraints to foraging, including thermoregulation and oxygen consumption, likely exacerbated her already poor condition. These findings improve our understanding of illness in free-ranging air-breathing marine megafauna, demonstrate the vulnerability of individuals at critical points in their life history, highlight the importance of considering individual health when interpreting biologging data and could help differentiate between malnutrition and other causes of at-sea mortality from transmitted data.

## Teaser

Disease is an important but poorly understood cause of mortality in wildlife. For the first time, we document behavioural and physiological changes due to illness in a free-ranging seal. The identified abnormalities are likely markers of disease and could help identify causes of mortality from remote tracking data.

## Introduction

There is considerable interest in understanding the consequences of stressors on vital rates in marine mammals by quantifying links between health, physiology, behaviour and fitness (Pirotta *et al.,* 2018; Keen *et al.,* 2021). The timing, magnitude and nature of stressors play a role in the long-term consequences for individuals and populations. Stressors during vulnerable stages of an animal's life history can be more detrimental than at other times in their cycle (Keen *et al.,* 2021). Despite its importance as a critical form of mortality, little is known about disease progression in air-breathing marine megafauna at sea. Standard biotelemetry devices provide detailed information about animal location and movement, but for individuals that never return from sea, information is limited to remotely transmitted data. These summary data are currently insufficient to differentiate between mortality due to predation, sickness, or malnutrition in part due to a lack of information about behavioural changes during illness. Implantable telemetry devices have been developed that enable researchers to identify mortality due to predation (Horning *et al.,* 2008; Horning and Mellish, 2012), but that approach is not applicable to externally attached devices. Detailed behavioural records of sick animals at sea are virtually non-existent but could provide vital information to (1) understand the consequences of at-sea sickness in a marine mammal; (2) provide a behavioural model to help researchers use transmitted data to differentiate between mortality due to deteriorating body condition and other, more abrupt, causes of mortality.

Infection is typically accompanied by fever, which helps slow the spread of pathogens (Kluger, 1978; Evans *et al.,* 2015), and a suite of associated behaviours, including inactivity, sleepiness and anorexia (Hart and Hart, 2019). These behaviours are consistent across vertebrate taxa and are viewed as an adaptive response, ensuring that body resources are conserved to fight the infection. Fever results from an increased thermoregulatory set point (controlled by cytokines). Animals feel chilly under conditions that would previously have felt normal. This results in the animal minimizing heat loss and generating sufficient body heat to maintain the elevated set-point, usually 1°C to 4°C above normal core body temperature (Hart and Hart, 2019). Heat loss is reduced through behavioural and physiological means, such as reducing movement, seeking warm places and constricting blood flow to the extremities. In marine mammals, these typical behavioural responses are confounded when an animal is at sea—in a cold environment where sedentary behaviour would leave them particularly vulnerable to predation.

Northern elephant seals (*Mirounga angustirostris*) have a demanding lifestyle, which includes two extended fasting periods each year in conjunction with other physiologically stressful processes (i.e. lactation/breeding and catastrophic moulting) (Worthy *et al.,* 1992; Fowler *et al.,* 2016). Their foraging behaviour is also physiologically demanding. While at sea, they dive continuously with minimal post-dive surface intervals (PDI ~ 2 minutes) to replenish oxygen stores used during 20 to 40 minutes dives (Le Boeuf *et al.,* 2000; Robinson *et al.,* 2012). These animals regularly dive at or near their calculated aerobic dive limit (Hassrick *et al.,* 2010) and are well adapted to withstand hypoxic conditions (Meir *et al.,* 2009; Meir *et al.,* 2013; Tift and Ponganis, 2019).

Adult female elephant seals from the colony at Año Nuevo State Park have been tracked and monitored continuously during both their post-breeding (March–May) and post-moulting (June–January) foraging trips since 2004 (Robinson *et al.,* 2012; Holser, 2020). While most animals (90.7% post-breeding and 84.2% post-moulting (Holser *et al.,* 2021)) return to Año Nuevo or another colony, a few animals disappear and presumably die during their time at sea (Kienle *et al.,* 2022). In February 2017, an outwardly healthy individual (6018) was sedated and instrumented for tracking at the end of the breeding season. She returned to the colony to moult in May 2017 in visibly poor condition, a unique occurrence among the 664 adult females that have been instrumented since 2004. As part of our standard protocol, we collected data on the individual's movements, body condition and clinical health parameters. Comparing this female’s data to our extensive control data provided a remarkable opportunity to examine the behaviour and physiology associated with disease in a freely ranging marine mammal. Our findings could help inform the interpretation of biologging data in terms of differentiating between causes of mortality and provides insight into the energetic consequences of an extended disturbance.

## Methods

### Study site and ethics

This study was conducted at the northern elephant seal colony at Año Nuevo State Park, San Mateo County, California, USA. All animal handling was conducted under NMFS permits 786-1463, 87-143, 14636 and 19108 and with the approval and oversight of the UCSC Institutional Animal Care and Use Committee.

### Field procedures

Adult female diving and foraging behaviour was measured using biologging instruments deployed on ~ 20 individuals per year during the post-breeding trip from 2004 to 2020 for a total of 289 deployments. These deployments are part of a long-term monitoring effort to evaluate how this species’ foraging behaviour changes in response to oceanographic conditions. Following best practices (Horning *et al.,* 2019), animals for this work are selected to maximize survival and return instruments with high-resolution data. We choose animals based on age (5–12 years), prior reproductive and moulting history and health from a visual assessment of the individual (i.e. no open wounds, clear eyes and skin, typical body condition). Elephant seals nurse their pups up until their departure for sea, so deployments are completed during late lactation, typically on Day 23 to Day 25 of a ~ 26-day lactation period. Each animal was sedated following standard protocols (Robinson *et al.,* 2012) and equipped with a time-depth recorder (TDR) programmed to collect depth data at least every 8 seconds and with a satellite tag providing either Argos or GPS locations (Wildlife Computers, Seattle, WA, USA; Sea Mammal Research Unit, St. Andrews University, UK). Seal 6018 instrumented with a jaw accelerometer (Little Leonardo, Japan) that recorded prey capture events (Naito *et al.,* 2013) in addition to the 48 seals in our previous study (Adachi *et al.,* 2021). Upon returning to shore, individuals were sedated again for instrument recoveries.

Body composition and energy gain values were calculated using established methods (Robinson *et al.,* 2012). Morphometric measurements were collected during deployment and recovery sedations, including weight, blubber depths, lengths and girths. Body composition was calculated using the truncated cones method (Gales and Burton, 1987) and calibrated to body water measurements (Webb *et al.,* 1998). Blubber thickness was measured at six evenly spaced locations along the length of the body along a dorsal line and again along a lateral line (12 measurements total) using a backfat ultrasound meter (Scanoprobe, Ithaca, NY) at deployment and a Signos handheld ultrasound (Signostics, Thebarton, AUS) at recovery. Both instruments have previously been used to measure blubber thickness in elephant seals and produce comparable data. Mass was measured each time animals were sedated by suspending the seal from a hanging scale using a tripod and sling. To determine mass upon arrival and departure from the colony, measured masses were corrected to account for time spent onshore fasting before (Simmons *et al.,* 2010) and after measurements were taken:

(Eq. 1) \begin{align*}&\notag {MassCorrection}_{Br\mathrm{e} ed}\\ &\quad=\left(1.2209+0.01329\ast Mass\right)\ast {DaysFasting}_{Br eed} \end{align*}

This mass-specific correction was derived from females that were weighed repeatedly while on shore for previous studies (n = 82; Crocker, unpublished data). Departure from and arrival at the colony of each instrumented animal were determined from TDR or satellite records.

The surface area to volume ratio (SA/V) was calculated for post-breeding seals in 2017 using morphometrics measured at deployment and recovery. Each seal was represented geometrically by seven circular truncated cones from the ankle to the ears (divided at the same six locations along the length of the body where blubber thickness was measured) and a normal circular cone from the ears to the nose for the head of the seal. This method was chosen as the least likely to overestimate or underestimate the dimensions of the seal. Surface area and volume were calculated as the sum of the lateral surface areas and volumes, respectively, of each of these geometric shapes. For comparison to our best estimate methodology, a minimum and maximum SA/V were also calculated using differing geometric representations of the seal. The minimum estimate was calculated without the normal cone representing the head of the seal (only truncated cones from ankles to ears). The maximum estimate was calculated with the addition of normal circular cones for both the head (ears to nose) and tail (ankles to tail tip) to the truncated cones from ankles to ears.

### Laboratory procedures

Blood samples were collected from the extradural vein at deployment and recovery in untreated and EDTA vacutainers. An EDTA whole blood sample was sent to Antech Diagnostics for a blood chemistry panel immediately following the recovery procedure. The remaining samples were centrifuged at 4°C and 2400 rpm for 15 minutes, and samples were stored at −80°C until analysis.

Blood samples were analysed for markers of both innate (cytokines interleukin 6 (IL-6) and interleukin 1β (IL-1β)) and adaptive (immunoglobulins IgG, IgM and IgE) immune responses, stress and thyroid hormones (cortisol, thyroxine (T4), triidothyrione (T3), reverse triidothyrione (rT3)) and metabolites (glucose, blood urea nitrogen (BUN), and non-esterified fatty acids (NEFA)). Analyses were completed using commercially available kits summarized in Table 1. These immunoassays methods have been validated in northern elephant seals (Ortiz *et al.,* 2001; Champagne *et al.,* 2005; Fowler *et al.,* 2008; Champagne *et al.,* 2013; Ensminger *et al.,* 2014; Fowler *et al.,* 2016; Peck *et al.,* 2016; Northey, 2021).

**Table 1:** Assays used to measure analytes reported here

	Analyte	Sample	Assay	Manufacturer	Validation
Cytokine	IL-1β	Serum	Canine IL-1 beta ELISA	RayBio, Morcross, GA, USA	Peck *et al.,* 2016
Cytokine	IL-6	Serum	Canine IL-6 ELISA	RayBio, Morcross, GA, USA	Peck *et al.,* 2016
Immunoglobulin	IgG	Serum	Easy-Titer Human IgG	Fisher Easy	Peck *et al.,* 2016
Immunoglobulin	IgM	Serum	Easy-Titer Human IgM	Fisher	Peck *et al.,* 2016
Immunoglobulin	IgE	Serum	Canine IgE ELISA	GenWay Biotech Inc.,	Peck *et al.,* 2016
Hormone	Cortisol	Serum	DPC RIA	Siemens, Washington DC, USA	Ortiz *et al.,* 2001
Hormone	T4	Serum	RIA	Siemens, Washington DC, USA	Ensminger *et al.,* 2014
Hormone	T3	Serum	RIA	Siemens, Washington DC, USA	Ensminger *et al.,* 2014
Hormone	rT3	Serum	RIA	Alpco, Salem, NH, USA	Northey, 2021
Metabolite	Glucose	Plasma	YSI 2300 glucose autoanalyser	YSI Inc., Yellow Springs, OH, USA	
Metabolite	BUN	Plasma	Enzymatic colorimetric assay	Stanbio, Boerne, TX, USA	
Metabolite	NEFA	Plasma	Enzymatic colorimetric assay	Wako Diagnostics, Richmond, VA, USA	

IL, interleukin; Ig, immunoglobulin

### Data analysis

Data processing and statistical analyses were completed in R v4.2.1 and MATLAB R2021a. Tracking and diving data underwent processing and quality control prior to analysis. Location estimates were filtered and processed using the R package foieGras (Jonsen *et al.,* 2019; Jonsen *et al.,* 2020; Jonsen and Patterson, 2020). This software uses a state-space model to eliminate erroneous location points and interpolate between locations at one-hour intervals, generating a realistic track of the animal's movement. For each track segment, the distance between locations and distance from the colony were calculated using the MATLAB function lldistkm (Meyer *et al.,* 2022). This allowed us to calculate the total distance travelled and maximum distance from the colony.

Diving data were processed in MATLAB using a custom toolbox (IKNOS) to zero-offset correct TDR records, identify dives and calculate basic statistics (i.e. depth, duration, ascent and descent rates, bottom time, wiggles, post-dive interval) for each dive. All dive records were subsampled to 8-second intervals to compare statistics across all deployments. Geographic locations for each dive were determined using timestamps of dives and a linear interpolation between points of the foieGras-processed satellite track. Solar elevation was calculated for each dive using the MATLAB function SolarAzEl (Koblick, 2014). Daytime and nighttime dives were separated based on a solar elevation greater than or less than 0°, respectively. Individuals with highly benthic foraging behaviour have distinctly different dive depths, movement and foraging rates than pelagic individuals (Adachi *et al.,* 2021). For this reason, we excluded three benthically foraging individuals from all comparisons. Foraging and non-foraging dives were determined from jaw accelerometer data. The total number of jaw motion events per day was calculated for seal 6018 and 45 healthy seals exhibiting pelagic foraging deployed during 2011 to 2018. These data were processed following the methods detailed in Adachi *et al.* (2021).

We calculated means and standard deviations for foraging success and movement metrics, and grand means and root-mean-squared error (RMSE) for diving metrics in all other post-breeding females (N = 247) as well as healthy females deployed in 2017 (N = 7) for comparison to 6018 (Table 2). For diving metrics, we used ANOVA and Tukey’s post-hoc tests to evaluate differences between 6018 and other deployments. There can be year-to-year differences in elephant seal movement and behaviour caused by environmental variation (Crocker *et al.,* 2006; Holser, 2020). We therefore restricted the following diving behaviour comparative analyses to healthy individuals deployed during the same year and season as 6018, i.e. post-breeding foraging trip in 2017 (N = 7). As jaw accelerometer data are sparser than diving data (only a few seals per year carry jaw accelerometers in addition to TDRs), we chose to compare 6018’s jaw acceleration record to all other pelagic foraging post-breeding seals (N = 45). We examined the relationship between 1) dive depth and dive duration and 2) dive duration and PDI by calculating two-dimensional kernel densities from the complete dive records of healthy individuals. We examined changes in diving and foraging behaviour (i.e. dive duration, prey capture events and depth) as a function of day of trip by fitting GAM smoothing curves using ggplot2 (Wickham, 2016) to the dive records of healthy individuals combined and to seal 6018 for comparison. Finally, we calculated the number of jaw motion events per day as a function of the percent time the animal spent foraging (as in Fig. 3 from (Adachi *et al.,* 2021)) as well as the distribution of depths at which jaw motion events occurred.

**Table 2:** Foraging success and behaviour of 6018 compared with all other post-breeding seals and with other 2017 post-breeding seals

Trip Metric	PB2004–2020 (N = 247)	6018	PB2017 (N = 7)
Mass gain (kg)	86.7 ± 22.5	−49.1	87.5 ± 17.4
Mass gain rate (kg/d)	1.2 ± 0.3	−0.54	1.1 ± 0.2
% Mass gain	27.5 ± 8.0	−17.9	29.9 ± 7.0
Energy gain (MJ)	1663.6 ± 528.1	−1121.9	1424.0 ± 330.3
Energy gain rate (MJ/d)	22.3 ± 7.8	−12.4	18.4 ± 5.1
Departure %Adipose	28.2 ± 2.1	24.7	28.3 ± 1.2
Arrival % adipose	31.5 ± 2.5	18.3	29.6 ± 1.6
Deploy blubber depth (cm)	3.35 ± 0.76	2.55 ± 0.22	3.33 ± 0.45
Recovery blubber depth (cm)	3.79 ± 0.67	1.72 ± 0.20	3.51 ± 0.42
Trip duration (days)	75.6 ± 11.0	91	78.7 ± 8.6
Max distance (km)	2108 ± 562	2884	2041 ± 703
Total distance (km)	5099 ± 1293	6569	5579 ± 661
# Dives per trip	4502 ± 890	6857	4734.1 ± 552.0
# PDI > 4 min per trip	48.0 ± 63.2	209	34.1 ± 18.3
**Diving Metric**	**PB2004–2020 (N = 236)**	**6018**	**PB2017 (N = 8)**
Max depth (m)	1193.0 ± 171.6	**920**	1218.1 ± 138.9
Mean depth (m)	542.7 ± 146.6	**426.5 ± 166.4**	534.8 ± 149.6
Mean depth day (m)	588.6 ± 146.4	**445.9 ± 197.6**	568.2 ± 153.7
Mean depth night (m)	503.5 ± 130.1	**407.5 ± 130.2**	493.5 ± 127.9
Dive duration (min)	22.3 ± 4.8	**16.3 ± 3.9**	21.2 ± 4.2
Dive duration day (min)	24.5 ± 4.4	**17.9 ± 4.0**	23.2 ± 4.1
Dive duration night (min)	20.4 ± 4.2	**15.0 ± 2.9**	19.5 ± 3.4
Bottom time (min)	10.3 ± 4.3	**4.8 ± 2.6**	8.2 ± 4.1
Bottom time day (min)	11.2 ± 4.4	**4.8 ± 2.8**	9.8 ± 4.1
Bottom time night (min)	9.4 ± 3.9	**4.6 ± 2.3**	8.2 ± 3.2
Ascent rate (m/s)	1.26 ± 1.03	**1.05 ± 0.24**	1.19 ± 0.23
Ascent rate day (m/s)	1.26 ± 1.10	**0.98 ± 0.25**	1.18 ± 0.22
Ascent rate night (m/s)	1.26 ± 0.90	**1.10 ± 0.22**	1.19 ± 0.25
Descent rate (m/s)	1.33 ± 0.71	1.29 ± 0.43	1.29 ± 0.35
Descent rate day (m/s)	1.34 ± 0.63	**1.17 ± 0.47**	1.29 ± 0.34
Descent rate night (m/s)	1.33 ± 0.57	**1.43 ± 0.36**	1.30 ± 0.35
PDI (min)	2.1 ± 5.1	**2.6 ± 8.3**	2.1 ± 6.1
PDI day (min)	2.1 ± 4.2	**3.1 ± 10.4**	2.1 ± 5.4
PDI night (min)	2.1 ± 5.8	2.2 ± 6.0	2.1 ± 6.6

Mean ± s.d. is reported for all values for 6018 and Trip Metric values for PB2004–2020 and PB2017. Diving Metric values for PB2004–2020 and PB2017 are grand means ± RMSE. For diving metrics, values in bold are significantly different (ANOVA with Tukey’s post hoc) from both other females from all years and other 2017 females

## Results

**Figure 1:**
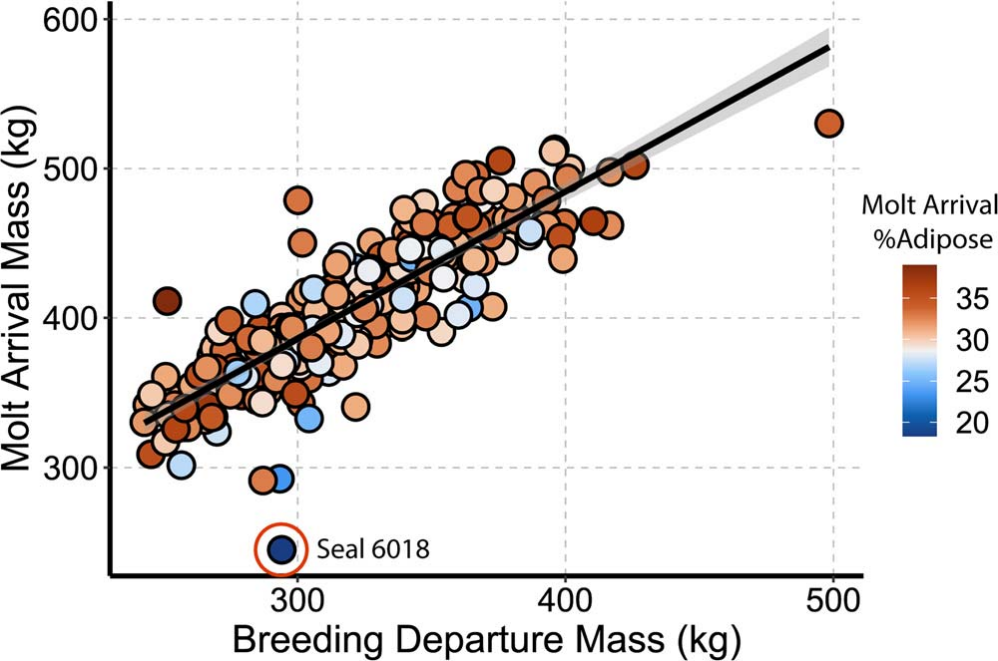
Arrival vs. departure mass (kg) for all post-breeding adult female elephant seals (N = 247). Colour indicates body composition at arrival on shore. The shaded line indicates the linear regression with 95% confidence interval for all individuals (R^2^ = 0.72).

Comparisons of 6018’s body condition, foraging success and at-sea behaviour relative to other animals measured across the 2017 post-breeding trip (N = 7) and to all post-breeding seals measured between 2004 and 2020 (N = 247) are summarized in Table 2. Seal 6018 was six years old at the time of deployment and had produced two pups during her lifetime, the first at age five. Her standard length was 253 cm, which is small for a 6-year-old (20^th^ percentile; 261 ± 9.4 cm, N = 88). At departure, her mass was 294 kg, which is typical for late lactation animals of that length (Supplementary S1), and her body condition was 24.7% adipose (73 kg adipose, 221 kg fat-free mass (FFM)). Upon return to shore, her mass had declined to 245 kg and 18.3% adipose (45 kg adipose, 200 kg FFM). This is the lowest body composition we have measured in an adult female northern elephant seal (Fig. 1).

**Figure 2:**
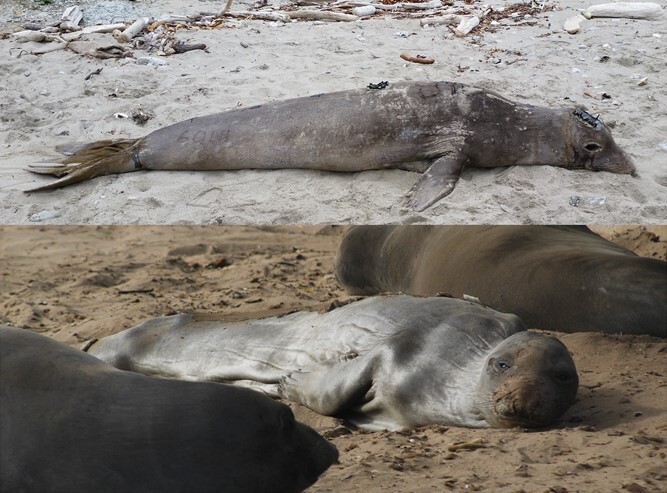
Photos of seal 6018 during her recovery sedation on 15 May 2017 (2 days after her arrival on shore) (top), and the last day she was seen 6 June 2017 (bottom).

Seal 6018 was lethargic at the time of recovery, and there were signs of infection on her flippers (Supplementary S2) and around a presumed cookie-cutter shark bite on her left side, mid-body, which were not present at deployment. She arrived on shore on May 13, 2017, for the moulting haul out and was sighted consistently for 25 days at the colony. While she was initially isolated from most other animals on the beach, she moved over half a mile to a larger group of hauled-out moulting animals within days. She began to moult visibly 14 days after arrival. At last sighting, approximately 70% of her body surface was moulted (Fig. 2B). She has not been resighted at the colony in the 5 years since and is presumed to have died at sea.

**Figure 3:**
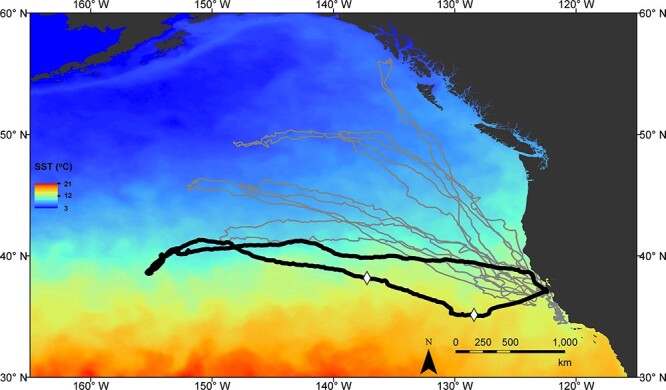
Map of all post-breeding 2017 tracks, overlaid on mean sea surface temperature (SST) for March 15—April 15. 6018 is shown here in black, with the start and end of her unusual dive behaviour (Days 8–20) indicated with diamonds. SST data are monthly 0.01° resolution from NOAA ERD and CoastWatch West Coast Regional Node (https://coastwatch.pfeg.noaa.gov/erddap/info/jplMURSST41mday/index.html).

Seal 6018 spent 91 days at sea during the post-breeding foraging trip prior to returning to moult. This is significantly longer than normal for a post-breeding trip (Table 2). Similarly long durations have been seen in otherwise healthy animals, particularly in years with poor foraging conditions (Crocker *et al.,* 2006). Tracking data show that her movement path was not unusual for an adult female elephant seal. However, she remained further south, in warmer water, than any of the other seals tracked post-breeding 2017 (Fig. 3). She travelled further from the colony and covered more total distance than is typical for a post-breeding trip (Fig. 3; Table 2), likely in part due to the additional 10 to 16 days she spent at sea.

**Figure 4:**
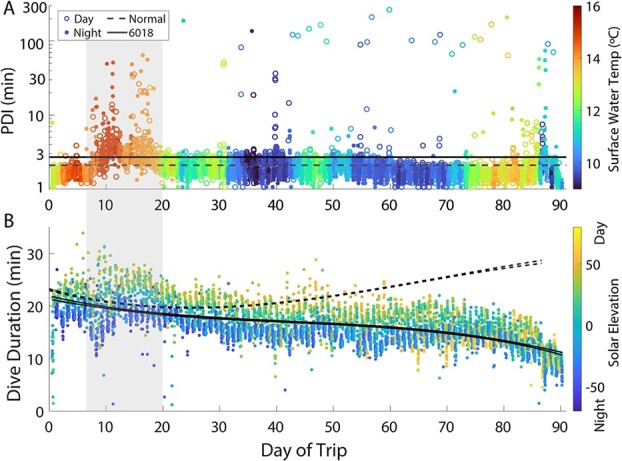
Diving characteristics of 6018 across her foraging trip. Shaded area indicates days 8–20. A) Post-dive intervals (minutes) following each dive during the daytime (open circles) and night-time (filled circles). Colour indicates the surface water temperature, measured in situ. B) Duration of each dive (minutes) in 6018’s TDR record. Colour indicates local time of day (solar elevation) at the start of each dive. The confidence intervals of fitted curves are represented by solid lines for 6018 and dashed lines for all other 2017 post-breeding animals.

**Figure 5:**
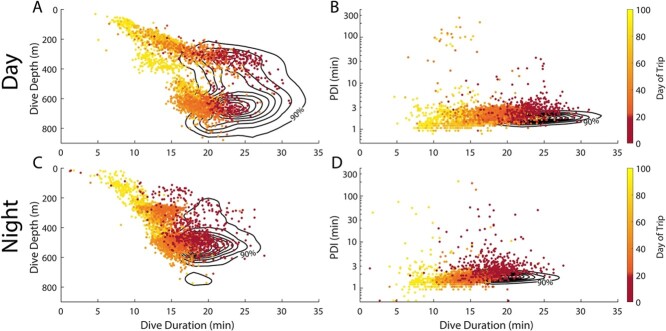
Diving characteristics of seal 6018 compared to typical adult female elephant seals. Daytime (A and B) and night-time (C and D) dive depth (m) and post-dive interval (minutes) in relation to dive duration (minutes). Contour lines indicate density distribution of all other post-breeding seals in 2017. Post-dive interval is presented on a log scale to accommodate the wide range of values observed. Each point represents a single dive in 6018’s dive record, with colour indicating the day of trip (red is the first 20 days of the trip and orange to yellow is from Day 20 on).

**Figure 6:**
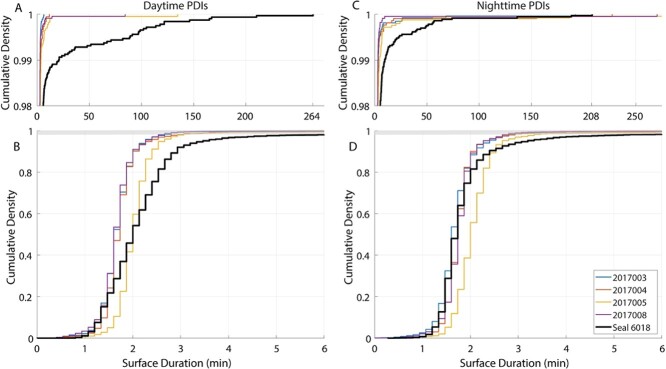
Cumulative contribution of different duration post-dive intervals during daytime (A and B) and night-time (C and D) in seal 6018 (black line) and other 2017 post-breeding seals (coloured lines). A and B are zoomed in to the top 2% of the cumulative density function (area highlighted with light grey in B and C) and show extended surface durations. The longest daytime surface interval recorded for 6018 was 264 minutes and the longest night-time PDI was 208 minutes.

During the first three weeks of the foraging trip, her diving behaviour displayed abnormal characteristics, particularly between Days 8 and 20. Her PDIs were consistently long (3–8 minutes) during this period, and her dives were a mixture of typical depth and duration and unusually short and shallow (Figs. 4 & 5). After the first three weeks, her PDI duration normalized; however, her dive duration declined over the remainder of the trip (Fig. 4). Her dives were often shallower than normal (Fig. 5A, C), and the bottom phase of her dives was half the duration of normal, healthy animals (Table 2). Although most of her PDIs were 1.5 to 4 minutes long, she still regularly exhibited extended surface intervals throughout the remainder of her trip, often during daytime hours (10–100 minutes; Fig. 4A & Fig. 6).

**Figure 7:**
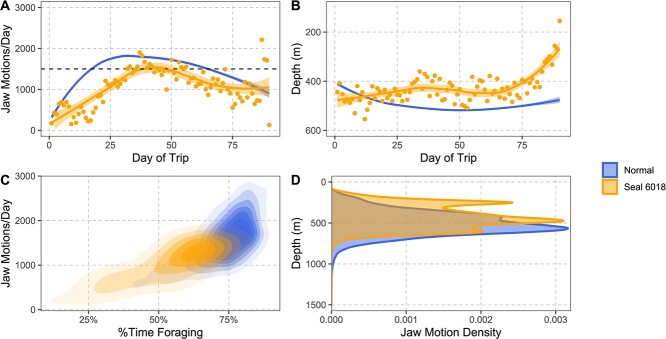
Spatial and temporal distribution of foraging during 6018's trip (orange) as compared with other pelagic foraging post-breeding seals (blue; N = 43). Panels A and B show GAM smoothers of total number of jaw motion events per day (A) and average depth of jaw motion events each day (B). The dashed line in panel A indicates the 1500 jaw motions per day threshold. Panel C shows the relationship between number of jaw motion events per day and percent time spent foraging. Panel D is the density distribution of the depth of jaw motion events, showing an unusual shallow peak in 6018's foraging effort between 200 and 300 m depth.

Compared to typical pelagic adult females, seal 6018 exhibited minimal foraging effort between Days 8 and 20 of her trip, with fewer than 500 prey capture attempts per day on all but 2 days. Typical females exhibit 1000 to 1500 prey capture attempts per day at the same stage of their trip (Fig. 7A). Across the entire trip, seal 6018 spent less of her time foraging than other pelagically foraging seals (Fig. 7C), and she foraged in shallower water (Fig. 7B, D).

A comparison of 6018’s physiological state to previous measurements in adult female elephant seals is summarized in Table 3. The results of the blood chemistry panel reported from the day of 6018’s recovery procedure are in Supplementary S3. Cytokine IL-1β was extremely elevated at both recovery and departure, and IL-6 was also elevated at recovery. Total IgG and IgM were both elevated at departure, but IgG and IgE were both suppressed at recovery. Her cortisol level was unusually high at deployment (15.4 μg/mL) and increased rather than decreased over the foraging trip (21.4 μg/mL at recovery). Thyroid hormones were within normal margins at departure, but at recovery T4 and T3 were both suppressed while rT3 was elevated. Blood glucose and BUN were elevated at recovery, while NEFA was low.

## Discussion

### Energetics and endocrine response

Adult female northern elephant seals need to forage nearly continuously (more than 1500 feeding events per day) on their post-breeding foraging trip. This is required to maintain a positive energy balance and regain some of the mass lost during the breeding fast (Adachi *et al.,* 2021). Seal 6018 only reached that feeding rate a few times during her trip (Fig. 7A). While most of the animals undergo a transit period, spending several weeks moving to richer foraging grounds, they do forage as they travel, typically exceeding 1000 prey capture events per day within ten days of departure (Fig. 7A). Seal 6018 was unable to cross that threshold until the fourth week of her post-breeding trip following her lactation fast, which contributed to her deteriorating condition.

Typical adult female elephant seals gain 1663.6 ± 528.1 MJ (Table 2) on their post-breeding trip. Seal 6018 lost 49.1 kg during her foraging trip (Table 2), 27.9 kg of adipose tissue and 21.0 kg of lean tissue. This is an energy deficit of 1090 MJ, of which 9% was derived from lean tissue (lean energy: 102.0 MJ = 21 kg * 27% water-free * 17.99 MJ kg^−1^; lipid energy: 987.6 MJ = 27.9 kg * 90% water-free * 39.33 MJ kg^−1^; (Pace and Rathbun, 1945; Worthy *et al.,* 1992; Schmidt-Nielsen, 1997; Crocker *et al.,* 2001)). Adult female elephant seals’ average field metabolic rate (FMR) during the post-breeding foraging trip is 90.1 kJ kg^−1^ d^−1^ (Maresh *et al.,* 2015). If seal 6018 had not foraged at all during the trip, she would incur an energy deficit of around 2212 MJ (given a departure mass of 294 kg, arrival mass of 245 kg, and assuming a linear decrease in mass across her trip), around double the 1090 MJ she did lose in body stores. Jaw motion data indicate that 6018 gradually increased foraging efforts after day 20 of her trip and video evidence shows that she foraged successfully on both fish and cephalopod prey items during the middle of her foraging trip (Yoshino *et al.,* 2020). These video loggers have relatively short lifespans, however, and do not allow us to quantify overall foraging success rates. While it is likely that her mass loss due is in part to poor foraging success during the trip, this explanation does not exclude the contribution of metabolic costs of illness to her declining body condition.

Mounting an immune response and maintaining an elevated body temperature are vital to fighting pathogen infections, but both are energetically costly (Brock *et al.,* 2013; Hart and Hart, 2019), especially for an animal immersed in water throughout the illness. In endothermic animals, there is a 10% to 12.5% increase in metabolic rate for every 1°C rise in body temperature (Evans *et al.,* 2015), and shivering can increase metabolic rate 2 to 3× resting level (Hart and Hart, 2019). A 4° fever could bring elephant seal FMR to 126.1–135.2 kJ kg^−1^ d^−1^ (assuming 10 and 12.5% increases, respectively). Combined with not foraging during the period of apparent illness early in her trip, this elevation would result in an energy cost of ~ 556 MJ over 14 days (15% of her trip), without accounting for shivering. This is roughly half of her total energy deficit, which underscores how elevated FMR and reduced foraging effort combined could account for dramatic mass loss. Reduced foraging effort during a febrile response would help maintain the inhospitably warm environment that slows pathogens (Kluger, 1978). Ingestion of cold prey causes a drop in stomach temperature of several degrees (Kuhn and Costa, 2006) which would be counterproductive to the febrile response. Compromising foraging and energy gains temporarily may be a worthwhile trade-off to maximize the effectiveness of the fever (and minimize its duration).

**Table 3:** Analytes measured from blood samples at instrument deployment (Late Breeding or LB) and recovery (Early Moult or EM), compared to mean values (± sd) in adult female elephant seals measured at the same life history stage

		6018		Population Means		
Analyte		Deploy	Recovery	Mean LB	Mean EM	Source
IL-6	(ng/mL)	0.44	**0.55**	0.45 ± 0.30	0.14 ± 0.10	Peck *et al.,* 2016
IL-1β	(pg/mL)	**231.5**	**76.4**	43.4 ± 7.7	21.6 ± 8.5	Peck *et al.,* 2016
IgM	(mg/mL)	**1.89**	0.23	0.41 ± 0.18	0.27 ± 0.05	Peck *et al.,* 2016
IgG	(mg/mL)	**16.68**	***0.78***	9.14 ± 5.25	7.15 ± 1.55	Peck *et al.,* 2016
IgE	(ug/mL)	**2.65**	***0.98***	1.96 ± 0.58	2.62 ± 0.92	Peck *et al.,* 2016
Cortisol	(ug/mL)	**15.4**	**21.4**	10.8 ± 3.6	3.0 ± 3.1	Peck *et al.,* 2016
T4	(ug/dL)	3.2	***2***	3.3 ± 1.1	5.2 ± 1.6	Crocker, unpublished
T3	(ng/dL)	143.5	***98.1***	162.7 ± 33.2	173.9 ± 23.6	Crocker, unpublished
rT3	(ng/mL)	2.4	**4.1**	1.9 ± 0.7	1.4 ± 0.6	Crocker, unpublished
glucose	(mg/dL)	120	**149**	119 ± 15	120 ± 12	Crocker *et al.* 2014, unpublished
BUN	(mg/dL)	***19***	**37**	22 ± 1	20 ± 2	Crocker *et al.* 2014, unpublished
NEFA	(mmol)	2.25	***0.78***	2.59 ± 1.12	1.00 ± 0.22	Fowler *et al.,* 2016

Values in **bold** are higher than normal for that life history stage. Bold ***italicized*** values are lower than normal.

Normally, metabolic depression occurs at the end of extended fasting periods in elephant seals (Rea and Costa, 1992), but mounting an immune response could override that normal energy conservation process. Elephant seals’ ability to spare protein as an energy source declines as their body composition decreases—at the end of the lactation fast, protein comprises 6–9% of metabolism (Crocker *et al.,* 1998). While 6018’s glucose and NEFA levels were normal at the end of lactation, her BUN and glucose were elevated at recovery. NEFA was relatively low (Table 3), consistent with high levels of protein metabolism even after her foraging trip. Combined, these metabolites likely indicate a shift away from fat-based metabolism that is characteristic of muscle energetics in phocids (Kanatous *et al.,* 2002). Seal 6018 also exhibited highly elevated cortisol and suppressed thyroid function at recovery, consistent with malnutrition (Tulp *et al.,* 1979; Armario *et al.,* 1987). Upregulating core body temperature, as needed for a febrile response, should elevate thyroid activity. However, after an additional ten weeks at sea following her apparent illness, this effect may not have been evident.

After arriving on shore, 6018 fasted for 25 days and was 70% moulted at last sighting. The mass-specific mass loss rate for a 245 kg animal, calculated as in Simmons *et al.* (2010), is 2.37 kg lost per day of fasting or ~ 59.3 kg lost at the end of 25 days, suggesting that seal 6018’s mass was approximately 189 kg (and below 18.3% adipose tissue) at the time she was last seen, and presumably departed to sea (Fig. 2B). Typically, moulted adult female elephant seals leave the colony at 282 ± 36 kg and 30.4 ± 2.6% adipose tissue (N = 252), having lost 137 ± 33 kg over a 43.3 ± 2.0 day fast (N = 16; Supplementary S4). Give her extremely low remaining body stores, seal 6018 was likely impelled to leave the colony in search of food before completing her moult.

### Immune response

Adult female elephant seals exhibit an elevated innate and adaptive immune response throughout lactation. Females with lower fat reserves at the end of lactation generally show reduced immune responses (Peck *et al.,* 2016). This individual, however, exhibited an unusual elevation in adaptive immune markers IgG and IgM prior to departure from the colony (Table 3) relative to typical late lactation females, indicating that she was already fighting an infection at the end of lactation. She also exhibited extremely high levels of IL-1β at departure, a cytokine that is released from the site of inflammation, helps battle infection and in conjunction with IL-6 can induce fever and alter animal behaviour, including reducing food intake and movement (Harden *et al.,* 2008). Cytokines can also induce the production of haptoglobin (Wang *et al.,* 2001; Peck *et al.,* 2016), which inhibits the microbial uptake of iron by scavenging free haemoglobin and thereby restrains pathogen growth (Hart and Hart, 2019). IL-6 is a pyrogenic cytokine that is heavily involved in inflammatory responses and plays multiple roles in an organism’s response to infection (i.e. pro-inflammatory, anti-inflammatory and repair-oriented) (Evans *et al.,* 2015; Del Giudice and Gangestad, 2018). When the animal returned to shore, her IL-1β level had dropped but was still significantly elevated relative to normal, as was IL-6. This continued cytokine elevation could reflect both illness during her foraging trip and damaged tissue (e.g. infected cookie-cutter shark bite, skin on flippers) in need of repair. While it is not uncommon for adult female elephant seals to return for the moult with one or two small sores on or around their flippers, the degree of infection observed on 6018 was unusual (Supplementary S2) and is likely symptomatic of her deteriorated condition.

The adaptive immune response is energetically costly and deteriorates if food is limited (Martin 2nd *et al.,* 2007). The adaptive immune markers IgG and IgE were both unusually low for seal 6018 at recovery (Table 3). Maintaining high IgG levels requires mobilizing substantial amounts of protein from lean tissue (Peck *et al.,* 2016), which the animal likely could not maintain given her depleted body stores and low foraging effort (she lost a further 21.0 kg of lean tissue after the lactation fast). Additionally, IgE is associated with helminth infection (parasitic worms) in elephant seals and is highest when they return from foraging. The low levels in this female may reflect reduced foraging or suppression from her high cortisol, as has been demonstrated previously in adult females (Peck *et al.,* 2016). Her low levels for these adaptive immune markers suggest that seal 6018 had become immunocompromised by the end of her foraging trip.

### Buoyancy, oxygen stores and thermoregulation

Body composition serves multiple roles in aquatic organisms—energy stores, insulation and buoyancy control. At departure, 6018 had a mass of 294 kg and an average blubber thickness of 2.55 +/− 0.22 cm. At recovery these values had decreased to 245 kg and 1.72 ± 0.20 cm blubber thickness (Table 2). This dramatic mass loss and blubber thickness reduction would simultaneously increase the animal’s locomotor costs, reduce her available oxygen stores and compromise her thermoregulation ability (Rosen *et al.,* 2007).

**Figure 8:**
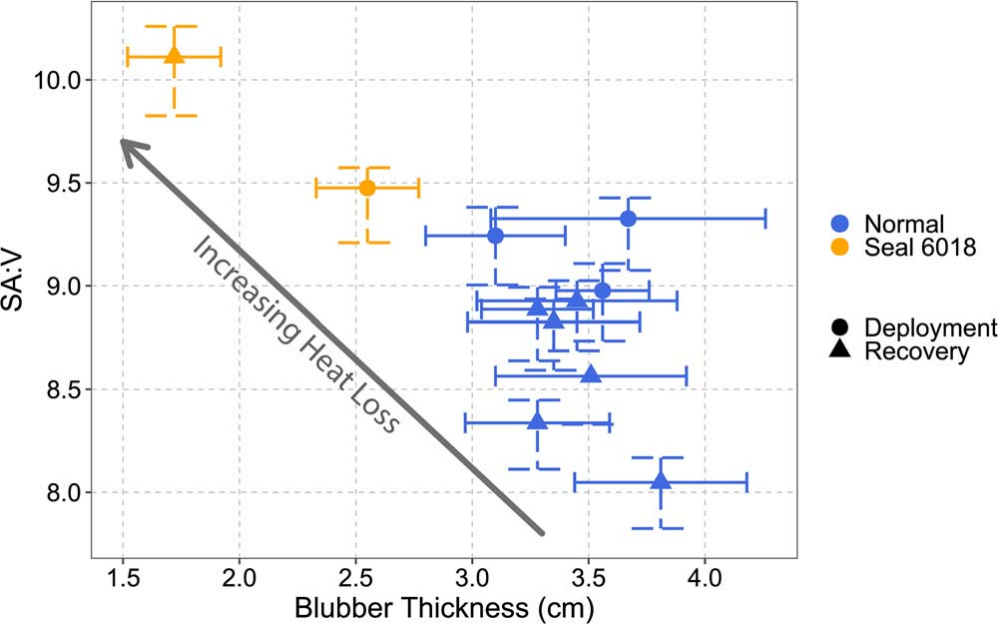
Surface area to volume ratio (SA/V) and average blubber thickness (cm) of 6018 and other 2017 post-breeding females. X-axis error bars indicate ±1 sd from the mean. Y-axis bars indicate SA/V calculated from tail to nose (max) and from ankles to ears (minutes) with the centre symbol indicating SA/V calculated from ankles to nose. Increasing SA/V results in greater heat loss to the environment, while decreasing blubber thickness reduces the animal’s insulation, also resulting in greater heat loss to the environment.

Elephant seals are typically negatively buoyant at departure from the colony. Their buoyancy gradually increases throughout their trip as they increase their proportion of lipid to lean body stores, allowing them to ascend from depth with less effort and lower metabolic costs (Adachi *et al.,* 2014; Maresh *et al.,* 2015). The optimum condition for energy-efficient locomotion is neutral buoyancy, which is likely achieved at a body composition of around 40% adipose (Webb *et al.,* 1998); both positive and negative buoyancy have greater metabolic costs (Adachi *et al.,* 2014). The body condition of 6018 decreased (from 24.7% to 18.3%) from the start to end of her trip, causing her locomotor efficiency to also decrease as she moved further away from neutral buoyancy (towards negative buoyancy) and well outside of the normal body condition range for adult female northern elephant seals. This reduced efficiency is evident in the low ascent rates, minimal bottom time and overall decline in dive duration throughout her foraging trip (Table 2; Fig. 4B). The increased effort required for 6018 to surface would exhaust her oxygen stores more quickly (Maresh *et al.,* 2015). Furthermore, any metabolic increase required as part of a response to illness (either immune upregulation or maintaining fever) would also contribute to more rapid oxygen depletion (Demas *et al.,* 1997; Bonneaud *et al.,* 2003; Martin 2nd *et al.,* 2003; Brock *et al.,* 2013) and constrain diving ability. Interestingly, 6018 had numerous shallow dives with very low descent and ascent rates (~0.5–0.8 m/s) that we do not see in typical seals (Supplementary S5 & Supplementary S6). These low-activity dives may have provided an alternative to more typical (and deeper) passive drift dives that allow elephant seals to rest and process food (Crocker *et al.,* 1997; Mitani *et al.,* 2010), enabling 6018 to rest despite her inefficient body condition.

Oxygen stores in diving animals scale with body size, as does metabolic rate (Halsey *et al.,* 2006; Crocker and McDonald, 2022). Larger animals have greater oxygen stores and a lower mass-specific metabolism, making them more efficient divers who can sustain longer periods underwater. Blood and muscle are the two primary oxygen stores for deep-diving marine mammals (Halsey *et al.,* 2006; Crocker and McDonald, 2022). Both reservoirs decreased during 6018’s foraging trip. The blood volume in the northern elephant seal is 20.2% of body mass (Hassrick *et al.,* 2010), typically ranging from 66.2–84.1 L of blood from deployment to recovery. Assuming typical blood volume proportions for the species, this animal would have begun her migration with 59.4 L of blood and ended with 49.5 L. Similarly, she departed the colony with 221 kg FFM and returned with 200 kg FFM, a substantial reduction in muscle and other lean tissue. These declining oxygen stores, combined with increased metabolic costs from locomotion and illness, results in the declining dive duration we see in this animal (Fig. 4B).

Thermoregulatory demands could also constrain the diving behaviour of a compromised animal. Even in temperate oceans, deep-diving animals will spend extended periods at water temperatures below 5°C. In addition to having a thin blubber layer, 6018’s surface area to volume ratio (SA/V) was unusually high by the time she returned to shore (Fig. 8). Because a low SA/V reduces heat loss to the environment (Herreid 2nd and Kessel, 1967; Favilla and Costa, 2020), her unusually high SA/V combined with lower body mass and a decrease in insulative capacity could limit the animal’s ability to sustain time spent at colder temperatures. The animal might have compensated behaviourally by spending more time at the surface in warmer water (particularly during daytime when solar radiation could contribute to warming). Many of 6018’s extended surface intervals (>10 minutes) took place during the day rather than at night, as is more typically seen in other individuals (Fig. 4A &6). While the duration of a surface interval can increase with dive duration, the extended PDIs seen in 6018’s record are unrelated to the duration of her dives (Fig. 5B, D). Spending time at the surface during the day would leave the seal more exposed to predators. Still, the need for warmth may have overridden that risk for this individual. This shows that disease, in addition to compromising an individual’s nutritional and physiological state, can induce high-risk behaviour, increasing overall vulnerability on multiple fronts.

## Conclusion

This study provides rare and valuable information about the progression and effects of disease in a deep-diving marine mammal. Bio-logging studies generally target healthy individuals and aim to generalize findings to the entire population of healthy individuals. The extremely poor physical conditions of 6018 at instrument recovery indicated poor health. This prompted a revaluation of her condition at deployment (via blood analytes and morphometrics) and a fine-scale examination of her diving behaviour. At deployment, the body condition of 6018 was typical of adult female elephant seals at the end of lactation (Supplementary S1) and her outward appearance was of a healthy seal. However, it is likely that she was already fighting an infection at the time. At sea, seal 6018 exhibited a clear, distinct behavioural response to this infection that aligns with the stereotypical behaviour of vertebrate animals during fever: she reduced her activity, spent more time in a warmer environment and severely reduced her food intake. These characteristics of her diving behaviour will inform the interpretation of transmitted diving data from animals lost at sea, helping researchers differentiate between body condition-related mortality and other causes.

Unfortunately, the energetic cost of this infection, which began concurrently with the end of the lactation fast, was high enough that 6018’s body condition declined beyond the point of recovery. Low body condition can compromise a marine mammal’s ability to dive long enough to forage adequately due to both thermoregulatory and oxygen limitations. Seal 6018’s diving performance continued to deteriorate over the remainder of her trip. She returned to shore in the poorest body condition recorded for a female northern elephant seal. Four weeks later, she left the colony prior to completing her annual moult and presumably died at sea. This illness occurred at a point of extreme vulnerability in the animal’s life-history; when she had minimal reserve body stores to sustain herself and combat the illness. The post-breeding foraging trip is a brief but critical window for adult female elephant seals to replenish body stores to fuel the moulting fast. Inadequate foraging success during this trip could compromise their ability to reproduce or even survive the next annual cycle. Further, the link between physiological state and behaviour should be carefully considered when evaluating the consequences of a stressor; a compromised state can cause high-risk behaviour. This sequence of events illustrates the importance of timing and context in determining the consequences of a stressor or disturbance, including disease.

## Funding

This work was supported by the Office of Naval Research [N00014–18-1-2822 and N000014–13-1-0134 to D.P.C and D.E.C.]; and the Strategic Environmental Research Development Program [RC20-C2–1284 to D.P.C and D.E.C.]; and the National Science Foundation [1644256 to D.P.C and D.E.C. and 1537203 to D.P.C]; and the Japan Society for the Promotion of Science [Grant-in-Aid for Scientific Research: 23255001 & 15 K14793; Grant-in-Aid for JSPS Fellows: 12 J04316 & 16 J02935; Grant-in-Aid for Research Activity Start-up: 15H06824]; and the Tagging of Pacific Predators Program including support from the Gordon and Betty Moore Foundation, the David and Lucile Packard Foundation, Alfred P Sloan Foundation and the Animal Telemetry Network.

## Supplementary Information

The Supplementary Materials file includes four figures and two tables (Supplementary S1-S6) that show 1) the relationship between mass and standard length of post-breeding deployment female elephant seals, 2) a photograph of the hind flippers of seal 6018, 3) the blood chemistry results from 6018’s recovery sample, 4) across-moult changes in body composition and length of time onshore for animals tracked before and after the moult in the same year, 5) descent and ascent rate in all of 6018’s dives compared to a typical seal and 6) depth versus duration with ascent and descent rate in all of 6018’s dives compared to a typical seal.

## Author Contributions

RRH—Conceptualization, Methodology, Software, Formal Analysis, Investigation, Data Curation, Writing—Original Draft, Writing—Review & Editing, Visualization, Project Administration.

DEC—Methodology, Validation, Resources, Investigation, Writing—Review & Editing, Funding Acquisition.

AF—Formal analysis, Investigation, Writing—Original Draft, Writing—Review & Editing.

TA—Methodology, Formal analysis, Investigation, Data Curation, Writing—Review & Editing.

TRK—Methodology, Formal analysis, Investigation, Data Curation, Writing—Review & Editing.

YN—Investigation, Resources, Writing—Review & Editing, Supervision, Funding Acquisition.

DPC—Investigation, Resources, Writing—Review & Editing, Supervision, Funding Acquisition.

## Conflicts of Interest

The authors have no conflicts of interest to declare.

## Data Availability

Foraging success data are available at the Dryad Digital Repository https://doi.org/10.7291/D1W101, tracking and diving data are available at the Dryad Digital Repository https://doi.org/10.7291/D18D7W and jaw motion data are available at the ADS (Arctic Data archive System) of the National Institute of Polar Research (https://ads.nipr.ac.jp/dataset/A20210316-001). Code used in data analysis are available at GitHub (https://github.com/rholser/EsealDiseaseAtSea) and Zenodo (http://doi.org/10.5281/zenodo.7864665).

## Supplementary Material

Web_Material_coad034
