# Supplementary material for: Effects of disease on foraging behaviour and success in an individual free-ranging northern elephant seal
Source: Conserv Physiol. 2023 May 25;11(1):coad034. doi: 10.1093/conphys/coad034 (PMC10214463; doi:10.1093/conphys/coad034)
Supplement: Web_Material_coad034 [file web_material_coad034.zip › DiseaseAtSea_ConsPhys_SI_Revision1.pdf]

# Supplementary Materials for

## Effects of disease on foraging behaviour and success in an individual free-ranging northern elephant seal

Rachel R. Holser*[1], Daniel E. Crocker[2], Arina Favilla[3], Taiki Adachi[3,4], Theresa R. Keates[5], Yasuhiko Naito[4], Daniel P. Costa[1,3]

1. Institute of Marine Sciences, University of California Santa Cruz, 115 McAllister Way, Santa Cruz, CA, 95060, USA

2. Department of Biology, Sonoma State University, Rohnert Park, California, 94928, USA

3. Department of Ecology and Evolutionary Biology, University of California Santa Cruz, Santa Cruz, California, 95064 USA

4. National Institute of Polar Research, Tachikawa, Tokyo, Japan

5. Department of Ocean Sciences, University of California Santa Cruz, Santa Cruz, California, 95064, USA

*Corresponding author

Phone: +1 253-514-0110

Email: rholser@ucsc.edu

ORCID: 000-0002-8668-3839

**This Supplementary Materials file includes four figures and two tables (S1-S6) that show 1) the relationship between mass and standard length of post-breeding deployment female elephant seals, 2) a photograph of the hind flippers of seal 6018, 3) the blood chemistry results from 6018's recovery sample, 4) across-moult changes in body composition and length of time onshore for animals tracked before and after the moult in the same year, 5) descent and ascent rate in all of 6018's dives compared to a typical seal, and 6) depth versus duration with ascent and descent rate in all of 6018's dives compared to a typical seal.**

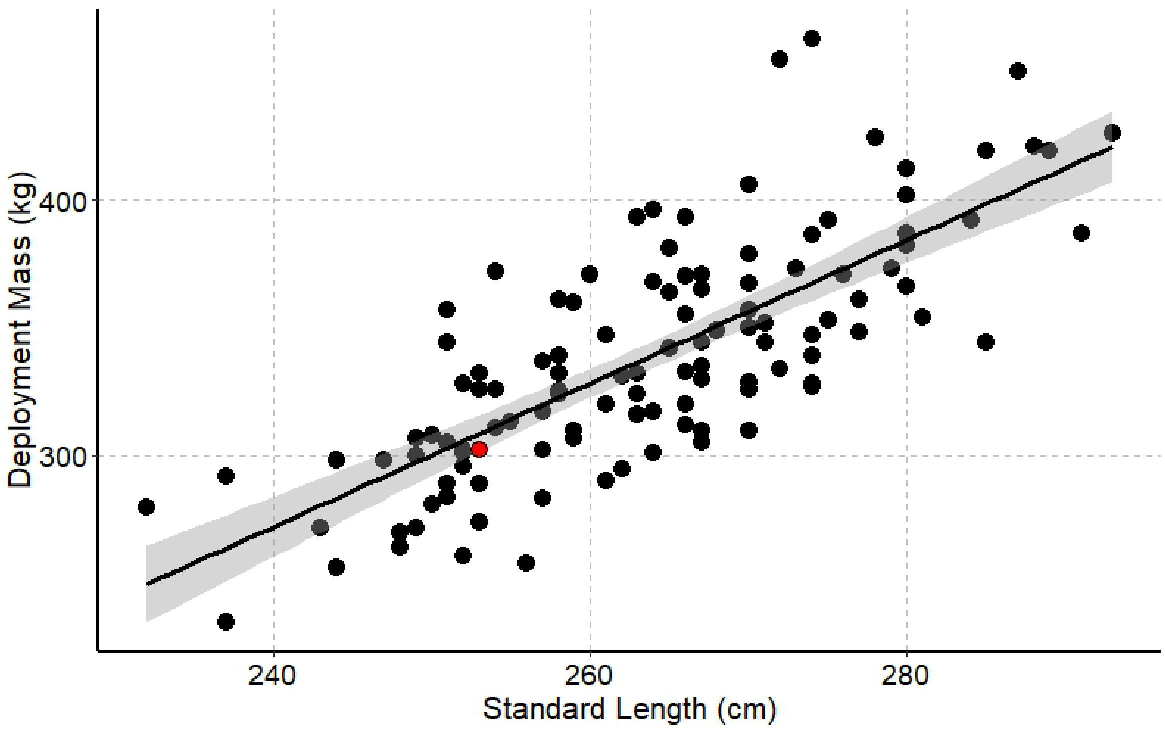


S1 – Mass (kg) and standard length (cm) of adult female elephant seals during post-breeding deployment procedures from 2013-2020 with a linear regression line and shaded 95% confidence intervals (N=118; $R^2$ = 0.578). Seal 6018 is shown in red.

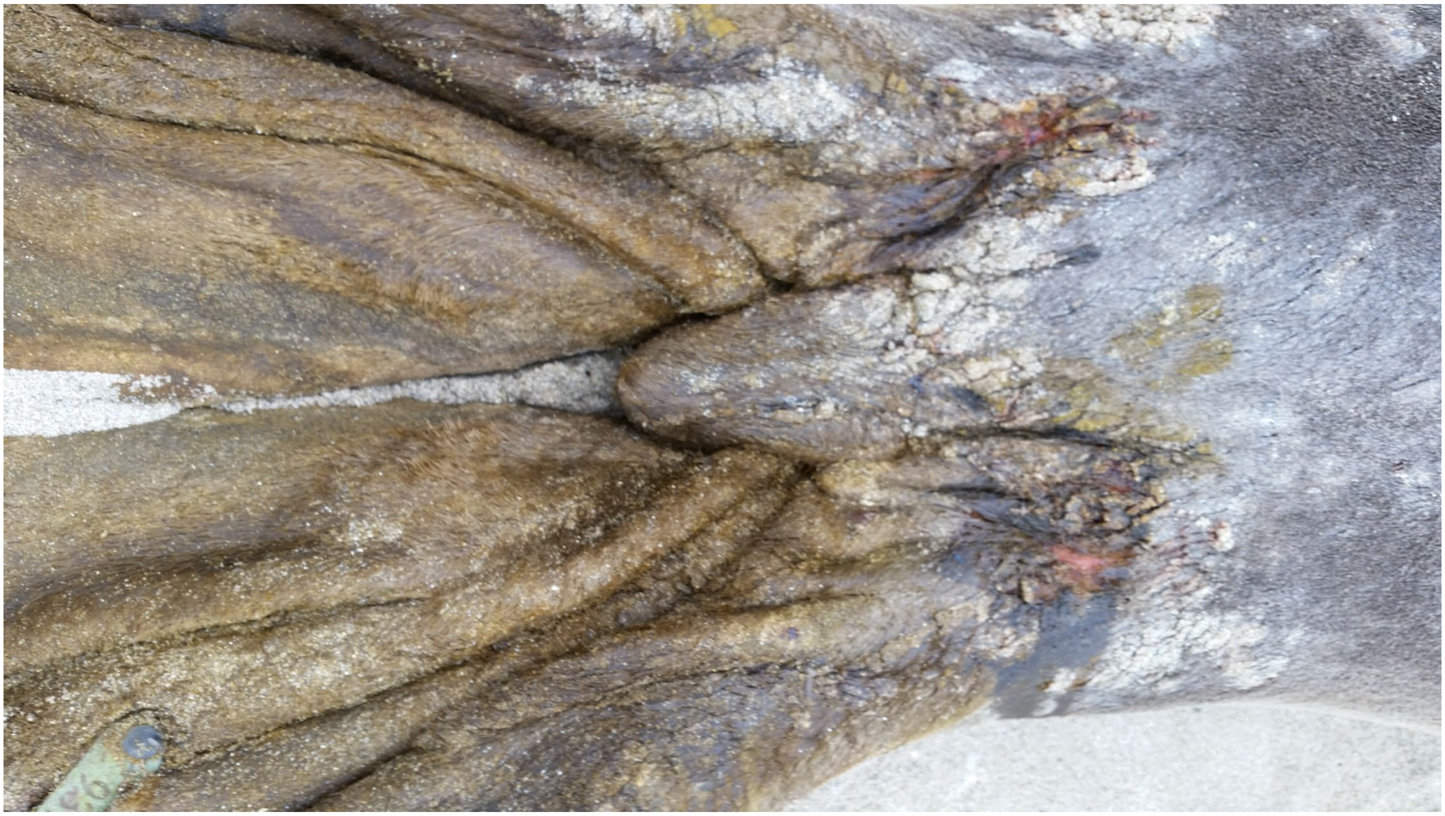

S2 – Photo of hind flippers during the recovery procedure, showing open cracks in her skin and signs of infection.

S3 – Results of blood chemistry analysis from 6018's recovery sample, completed by Antech Diagnostics (USA). Reference levels calculated from early moult adult females and males (N = 49; Crocker, unpublished).

| **Test** | **Results** | **Reference Levels** |
|---|---|---|
| Total Protein | 7.5 g/dL | 7.3 ± 0.4 |
| Albumin | 2.7 g/dL | 3.2 ± 0.2 |
| Globulin | 4.8 g/dL | 4.0 ± 0.4 |
| A/G Ratio | 0.6 | 0.8 ± 0.1 |
| AST (SGOT) | 47 IU/L | 15.0 ± 5.6 |
| ALT (SGPT) | 24 IU/L | 15.2 ± 3.4 |
| Alk Phosphatase | 136 IU/L | 262.5 ± 252.7 |
| GGT | 23 IU/L | |
| Total Bilirubin | 1.0 mg/dL | 0.6 ± 0.2 |
| BUN | 37 mg/dL | 31.6 ± 10.6 |
| Creatinine | 0.8 mg/dL | 1.8 ± 0.4 |
| BUN/Creatinine Ratio | 46 | |
| Phosphorous | 6.0 mg/dL | 6.5 ± 0.6 |
| Glucose | 149 mg/dL | 136.5 ± 18.3 |
| Calcium | 8.6 mg/dL | 9.6 ± 0.5 |
| Magnesium | 2.0 mEq/L | |
| Sodium | 140 mEq/L | 140.5 ± 3.3 |
| Potassium | 4.7 mEq/L | 4.3 ± 0.3 |
| Na/K Ratio | 30 | 32.9 ± 2.0 |
| Chloride | 99 mEq/L | 99.2 ± 2.8 |
| Cholesterol | 293 mg/dL | 367.3 ± 59.2 |
| Triglyceride | 58 mg/dL | 106.4 ± 50.3 |
| Amylase | 61 IU/L | |
| PrecisionPSL | 14 U/L | |
| CPK | 752 IU/L | 824.7 +/- 505.3 |
| Comments | | |
| Hemolysis 1+ | No significant interference | |

S4 – Changes in body composition and duration of time onshore during the moult in all animals tracked during both PB and PM the same year. Fat free mass (FFM) calculated assuming 10% water content in adipose tissue.

| SealID | Age | Time On Shore | Early Moult %Adip | Early Moult Mass (kg) | Late Moult %Adip | Late Moult Mass (kg) | Early Moult FFM (kg) | Late Moult FFM (kg) | FFM Lost (kg) | %FFM Lost | Mass Lost (kg) | Mass Loss /Day | %Mass Lost Lean | %Mass Lost |
|---|---|---|---|---|---|---|---|---|---|---|---|---|---|---|
| 1914 | 4 | 44.9 | 32.0% | 341.9 | 30.7% | 229.5 | 243.4 | 166.1 | 77.3 | 31.7% | 112.4 | 2.50 | 68.7% | 32.9% |
| N410 | 8 | 43.0 | 31.3% | 386.0 | 26.8% | 257.7 | 277.3 | 195.6 | 81.7 | 29.5% | 128.3 | 2.98 | 63.7% | 33.2% |
| O585 | 7 | 42.0 | 32.5% | 394.8 | 28.0% | 284.4 | 279.4 | 212.8 | 66.6 | 23.8% | 110.4 | 2.63 | 60.4% | 28.0% |
| R999 | 10 | 39.8 | 32.8% | 419.8 | 30.2% | 298.6 | 295.8 | 217.4 | 78.4 | 26.5% | 121.1 | 3.04 | 64.8% | 28.9% |
| T35 | 6 | 43.7 | 33.2% | 423.9 | 31.0% | 286.6 | 297.2 | 206.8 | 90.4 | 30.4% | 137.2 | 3.14 | 65.9% | 32.4% |
| 1317 | 10 | 43.2 | 31.9% | 437.7 | 26.9% | 232.1 | 311.9 | 175.9 | 135.9 | 43.6% | 205.5 | 4.76 | 66.1% | 47.0% |
| 4534 | 4 | 42.5 | 29.2% | 367.8 | 28.1% | 250.8 | 271.3 | 187.5 | 83.8 | 30.9% | 117.0 | 2.75 | 71.6% | 31.8% |
| U256 | 6 | 47.2 | 33.1% | 431.4 | -- | 265.9 | 302.8 | -- | -- | -- | 165.6 | 3.51 | -- | 38.4% |
| U481 | 6 | 41.3 | 31.3% | 361.0 | 28.2% | 235.3 | 259.4 | 175.6 | 83.9 | 32.3% | 125.8 | 3.04 | 66.7% | 34.8% |
| U848 | 6 | 44.8 | 32.1% | 433.7 | 26.8% | 293.4 | 308.4 | 222.6 | 85.8 | 27.8% | 140.3 | 3.13 | 61.1% | 32.3% |
| X349 | 5 | 35.6 | 31.2% | 368.3 | 27.9% | 252.7 | 264.7 | 189.3 | 75.5 | 28.5% | 115.6 | 3.24 | 65.3% | 31.4% |
| X387 |  | 43.7 | 33.7% | 389.6 | 28.1% | 256.1 | 271.4 | 191.2 | 80.2 | 29.5% | 133.6 | 3.06 | 60.0% | 34.3% |
| U448 | 7 | 43.6 | 31.2% | 417.3 | 29.6% | 283.8 | 300.2 | 208.1 | 92.0 | 30.7% | 133.5 | 3.06 | 69.0% | 32.0% |
| 5572 | 5 | 41.4 | 36.2% | 326.0 | 33.3% | 222.6 | 219.7 | 155.9 | 63.8 | 29.0% | 103.4 | 2.50 | 61.7% | 31.7% |
| 5842 | 4 | 40.8 | 33.7% | 385.9 | 29.3% | 253.7 | 268.8 | 186.8 | 82.0 | 30.5% | 132.2 | 3.24 | 62.0% | 34.3% |
| 9678 |  | 40.6 | 34.9% | 452.1 | 34.4% | 321.1 | 310.2 | 221.6 | 88.6 | 28.6% | 131.0 | 3.23 | 67.6% | 29.0% |
| Mean |  | 43.3 | 32.0% | 400.4 | 28.8% | 263.2 | 284.9 | 194.6 | 87.7 | 30.9% | 137.2 | 3.16 | 65.9% | 34.1% |
| StDev |  | 2.01 | 1.2% | 31.6 | 1.7% | 23.8 | 20.4 | 17.8 | 20.8 | 5.8% | 32.8 | 0.50 | 3.4% | 4.3% |

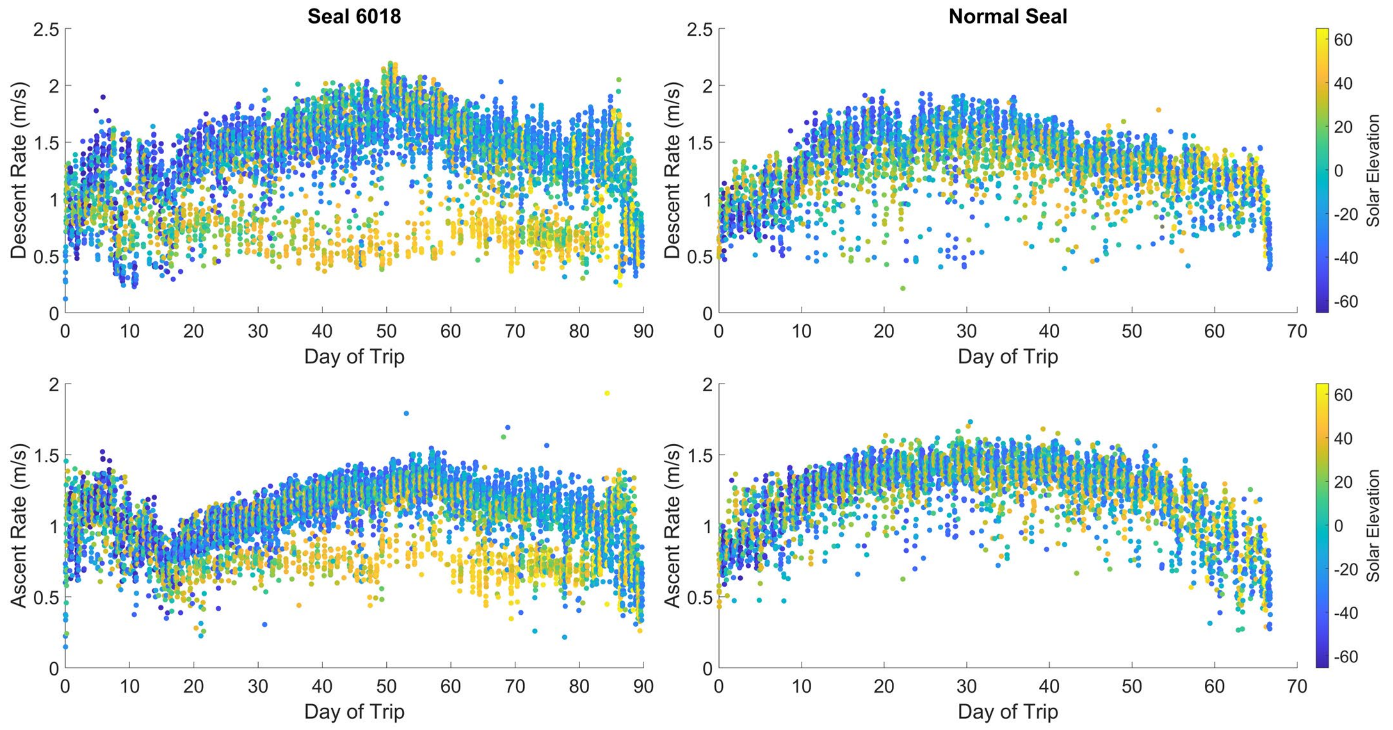


S5 - Descent and ascent rates of dives in seal 6018 and a normal seal from 2017 across the duration of their post-breeding foraging trip. Each dot represents a single dive and shows the average vertical rate of change for the ascent and descent phases of that dive. Color indicates solar elevation, with yellow colors as daytime and blues as night. Descent rates (m/s) are shown in panels A and B, ascent rates (m/s) in C and D.

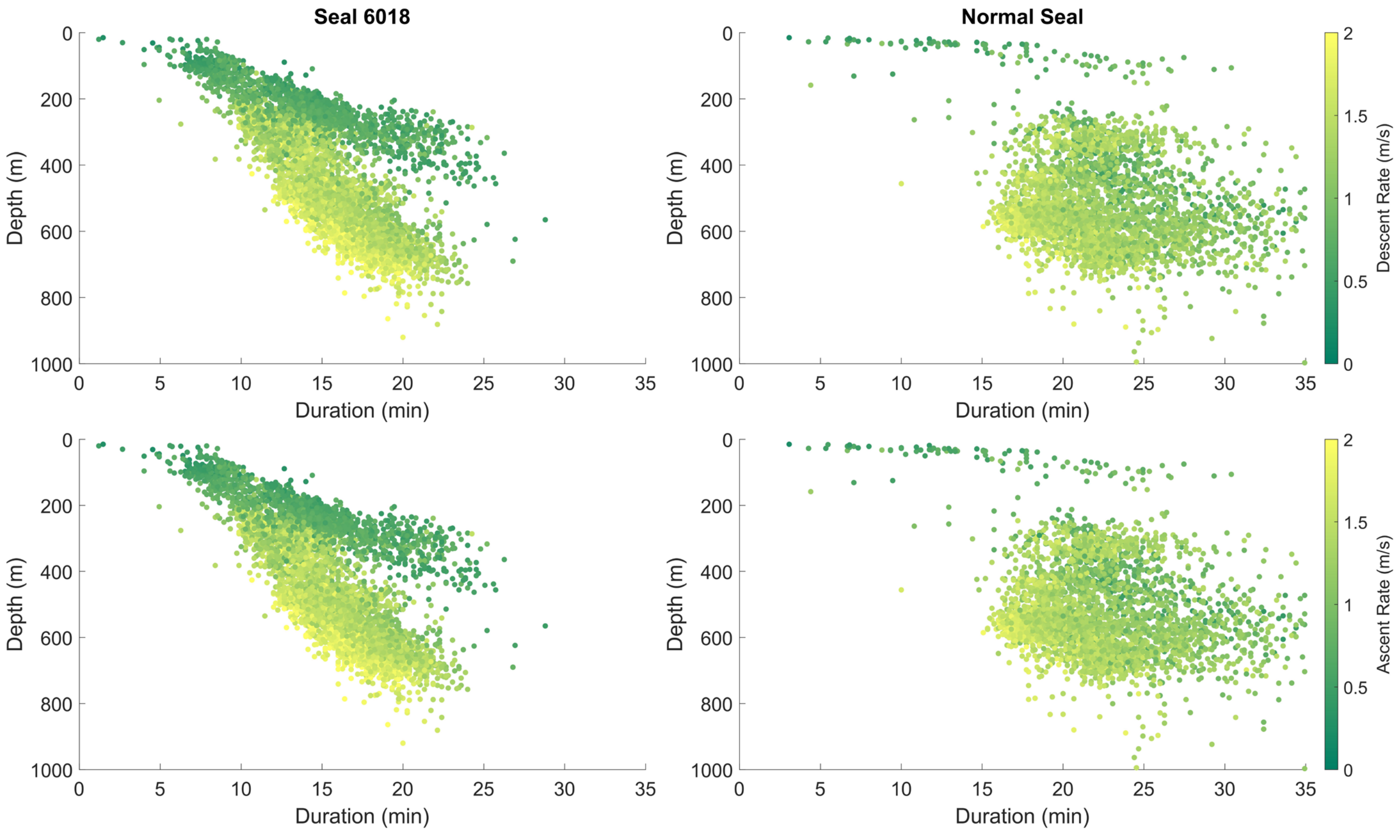


S6 – Depth and duration of dives in seal 6018 and a normal seal from 2017 with ascent and descent rate shown as color. Each dot represents a single dive and shows the maximum depth (m) and the total duration (min) of the dive, along with average vertical rate of change (m/s) for the ascent and descent phases of that dive, respectively. Darker greens indicate a slower rate of descent (panels A and B) or ascent (panels C and D).
